# Deciphering mineralogical changes and carbonation development during hydration and ageing of a consolidated ternary blended cement paste

**DOI:** 10.1107/S205225251701836X

**Published:** 2018-01-19

**Authors:** Francis Claret, Sylvain Grangeon, Annick Loschetter, Christophe Tournassat, Wout De Nolf, Nicholas Harker, Faiza Boulahya, Stéphane Gaboreau, Yannick Linard, Xavier Bourbon, Alejandro Fernandez-Martinez, Jonathan Wright

**Affiliations:** a BRGM, 3 avenue C. Guillemin, BP 36009, Orléans Cedex 2, 45060, France; b Université d’Orléans – CNRS/INSU-BRGM, UMR 7327 Institut des Sciences de la Terre d’Orléans (ISTO), Orléans, 45071, France; c Energy Geoscience Division, Lawrence Berkeley National Laboratory, 1 Cyclotron Road, Berkeley, CA 94720, USA; d ESRF, The European Synchrotron, 71 avenue des Martyrs, Grenoble, 38000, France; e Andra, Centre de Meuse/Haute Marne, Bure, 55290 France; f ISTerre, CNRS and University Grenoble Alpes, Grenoble, 38041, France

**Keywords:** cement, synchrotron radiation, X-ray diffraction tomography, carbonation, porosity, calcite

## Abstract

To understand the main properties of cement, a ubiquitous material, a sound description of its chemistry and mineralogy, including its reactivity in aggressive environments and its mechanical properties, is vital. In particular, the porosity distribution and associated sample carbonation, both of which affect cement’s properties and durability, should be quantified accurately, and their kinetics and mechanisms of formation known both in detail and *in situ*. However, traditional methods of cement mineralogy analysis (*e.g.* chemical mapping) involve sample preparation (*e.g.* slicing) that can be destructive and/or expose cement to the atmosphere, leading to preparation artefacts (*e.g.* dehydration). In addition, the kinetics of mineralogical development during hydration, and associated porosity development, cannot be examined. To circumvent these issues, X-ray diffraction computed tomography (XRD-CT) has been used. This allowed the mineralogy of ternary blended cement composed of clinker, fly ash and blast furnace slag to be deciphered. Consistent with previous results obtained for both powdered samples and dilute systems, it was possible, using a consolidated cement paste (with a water-to-solid ratio akin to that used in civil engineering), to determine that the mineralogy consists of alite (only detected in the *in situ* hydration experiment), calcite, calcium silicate hydrates (C-S-H), ettringite, mullite, portlandite, and an amorphous fraction of unreacted slag and fly ash. Mineralogical evolution during the first hydration steps indicated fast ferrite reactivity. Insights were also gained into how the cement porosity evolves over time and into associated spatially and time-resolved carbonation mechanisms. It was observed that macroporosity developed in less than 30 h of hydration, with pore sizes reaching about 100–150 µm in width. Carbonation was not observed for this time scale, but was found to affect the first 100 µm of cement located around macropores in a sample cured for six months. Regarding this carbonation, the only mineral detected was calcite.

## Abstract 2

Both a cured cement paste and a paste undergoing *in situ* hydration have been characterized using state-of-the-art synchrotron diffraction tomography. The mineralogy, macroporosity and carbonation phenomena of the samples have been determined.

## 1. Introduction   

How does cement, the most widely used industrial material in the world, interact with its environment? Understanding, quantifying and controlling cement-based material reactivity has profound implications for a wide range of industrial and environmental issues, such as modern building durability and sustainability (Ioannidou *et al.*, 2016 ▸), the overall integrity and long-term performance of a reinforcing structure (Kwon *et al.*, 2017 ▸), worldwide anthropogenic carbon dioxide production (Uwasu *et al.*, 2014 ▸), solar-powered desalination (Sellami *et al.*, 2016 ▸) and nuclear waste encapsulation/waste storage facility performance (Alonso *et al.*, 2010 ▸; Bildstein & Claret, 2015 ▸). Concrete has a long history that began in the pre-Roman age. One of the main improvements made to its formulation occurred at the beginning of nineteenth century, with the invention of Portland cement. Since that time, it has not changed significantly (Camoes & Ferreira, 2010 ▸), although the formulations have become more sophisticated [*e.g.* low-alkaline concrete (Lothenbach *et al.*, 2012 ▸) and alkali-activated slag binders]. On the other hand, the characterization techniques (Aranda, 2016 ▸) and associated mathematical formalisms of data analysis (Abdolhosseini Qomi *et al.*, 2014 ▸; Grangeon, Claret, Linard & Chiaberge, 2013 ▸) are constantly being improved so that we can better understand how this complex material changes over time.

Concrete is a composite material made of a porous matrix (the hydrated binder) filled with water, into which are embedded filler materials such as quartz and calcite, which act as a granular skeleton. As the hydration reaction proceeds due to the cement–water interaction, the anhydrous phases are converted into hydrates, leading to a decrease in the bulk porosity, since the molar volume of the hydrates is much larger than that of the anhydrous phases (Gaboreau *et al.*, 2017 ▸; Van Damme *et al.*, 2013 ▸). Porosity changes and mineralogical reactions can also occur after the initial hydration stage because of the reactivity of cement with its environment (*e.g.* well casing integrity during geological CO_2_ sequestration; Jun *et al.*, 2017 ▸).

Quantitative data on spatial distribution, modal content and associated formulae for each identified mineral and phase in the binder at a micrometre level of resolution can be determined using microprobe analysis (Gaboreau *et al.*, 2017 ▸). However, it is difficult to track the mineral and porosity changes occurring in real, dense pastes, relative to dilute samples (Van Damme *et al.*, 2013 ▸), and it is even more challenging to do so in both a non-invasive and time-resolved manner. The few non-invasive techniques that allow the *in situ* characterization of both the mineralogy and porosity during the reaction of cement materials are phase contrast tomography (PCT) and its variants (Prade *et al.*, 2015 ▸; Sarapata *et al.*, 2015 ▸). PCT is very useful, although it only provides indirect information about mineralogy. For example, it is difficult to distinguish between phases having a similar density and chemical composition. In contrast, X-ray diffraction computed tomography (XRD-CT) data contains structural information, allowing spatially resolved quantitative phase analysis. XRD-CT has been applied to pioneering studies to investigate hydration, nucleation mechanisms and microstructural development in cements (Voltolini *et al.*, 2013 ▸; Artioli *et al.*, 2015 ▸, 2010 ▸), but the evolution of cement porosity and the potentially associated carbonation mechanisms were not evaluated. In addition, no distinction was made between the nanocrystalline calcium silicate hydrate (C-S-H) phases and the X-ray amorphous fractions (*e.g.* fly ash that does not hydrate).

Here, taking advantage of our recent developments in C-S-H characterization that allow us to discriminate between this phase and the amorphous components of cement (Grangeon, Claret, Lerouge *et al.*, 2013 ▸; Grangeon, Claret, Linard & Chiaberge, 2013 ▸; Grangeon *et al.*, 2016 ▸, 2017 ▸), we present results obtained by synchrotron XRD-CT of a cement paste formulation consisting of blended Portland cement, fly ash and blast furnace slag (Chen *et al.*, 2012 ▸), as is expected to be used for nuclear waste disposal applications (Bildstein & Claret, 2015 ▸). By investigating short (24 and 30 h) and long (180 day) hydration periods, we were able to decipher the mineralogical changes and carbonation development during the hydration and ageing of a consolidated ternary blended cement paste.

## 2. Materials and methods   

### 2.1. Cement paste   

The composite cement used in this study was Rombas’s CEM V/A (Calcia), a blended cement with enhanced durability obtained by mixing about 50 wt% clinker with 25 wt% blast furnace slag (BFS) and 25 wt% fly ash (FA, mainly silica fume). A detailed composition is given in the supporting information (Table S1). This composite cement is suitable for subterranean work in harsh environments. Its CO_2_ emission footprint is also reduced, because the clinker is in part substituted by other constituents. A cement paste (a cylinder 8 cm high and 8 cm in diameter) was prepared using a water/binder ratio of 0.4 and then cured in a humidity chamber with a relative humidity >98% for approximately six months before being subjected to testing. The sample used for the measurements (a cylinder 1.6 mm in diameter and 1 cm high) was obtained by micro-drilling the larger sample. Another paste was prepared using the same water/binder ratio, and this was immediately transferred to a polyimide tube (1.3 mm in diameter) and subjected to testing after 24 and 30 h of hydration.

### 2.2. *In situ* XRD-CT measurements   

**Figure 1:**
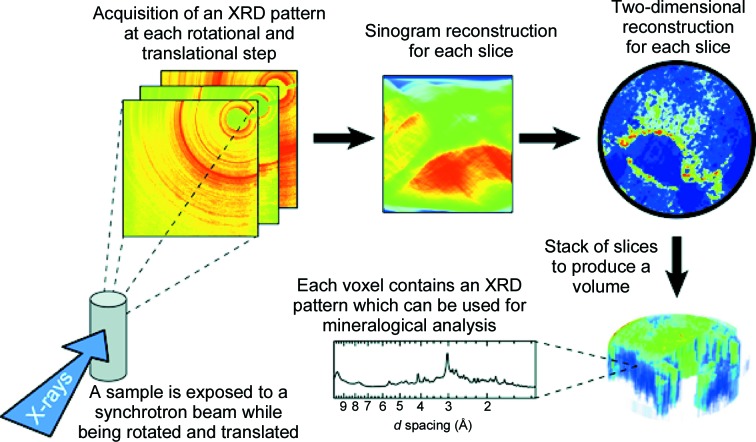
Schematic representation of an *in situ* synchrotron XRD-CT experiment. A slice corresponds to a height of 10 µm and a width of approximately 1.6 mm, *i.e.* 180 (rotation steps) × 160 (translation steps parallel to the beam) images. For the sample cured for six months, three slices were recorded (10 µm translation perpendicular to the beam). A sinogram represents the scattered intensity within one particular scattering angle.

XRD-CT measurements were performed on the ID11 beamline at the European Synchrotron Radiation Facility (ESRF, Grenoble, France). Fig. 1 ▸ shows the experimental scheme. A monochromatic incoming beam, tuned to an energy of 65.4 keV (λ = 0.1897 Å), was used to illuminate the samples. The instruments were calibrated using a NIST-certified CeO_2_ standard. The X-ray energy, combined with the selected sample-to-detector distance, covered a range of *d* spacings between 18.5 and 1.07 Å. The X-ray beam was focused with a Kirkpatrick–Baez mirror system, giving a beam size of 10 × 10 µm. As described in a previous study (Bleuet *et al.*, 2008 ▸), radial diffraction profiles (with a voxel size of 10 × 10 × 10 µm) were acquired in transmission mode with a two-dimensional fast-readout low-noise (FReLoN) detector while translating (along *y*, which is horizontal and perpendicular to the beam) and rotating the sample (along ω, with an angle of rotation about an axis which is vertical and perpendicular to the beam). Briefly, data were collected as follows. First, the sample was positioned to a given value of *y* which corresponded to a position slightly outside the sample. Then, data were collected at values of ω ranging from 0 to 180° in steps of 1°. Finally, control images were taken at ω values of 0°, 180° and 90°. Subsequently, the sample was translated along *y* by 10 µm and the recording along ω was repeated. Translations along *y* and data recording along ω were performed until the value of *y* corresponded to a point just outside the sample, on the opposite side to the start point. Successive azimuthal integrations gave a set of linear diffraction patterns, which were used to build a sinogram through the back Fourier transform of the data (De Nolf *et al.*, 2014 ▸). A 0.2 s exposure time was used to collect the data (for every value of the *y*, ω couple). A dark current of the same exposure time was also recorded after each data acquisition. The time needed to rotate the sample by 1° along ω (from 0° to 180°) was negligible but, in contrast, it took about 40 s to obtain the three control images for each *y* step (at precisely controlled values of ω of 0°, 90° and 180°). Translating the sample along *y* took about 10 s. Consequently, overall, this experimental setup allowed the recording of every slice of 10 µm thickness by 1.6 mm diameter in around 5.4 h.

### 2.3. Relevance of spatial resolution   

The spatial resolution used here (10 µm voxel size) is slightly poorer than that previously reported (4 µm voxel size) (Artioli *et al.*, 2015 ▸; Voltolini *et al.*, 2013 ▸). However, even a resolution of 0.5 µm does not allow us to take all the porosity of a cement paste into account (Zhang, 2017 ▸), mainly because concrete-based materials are multi-scaled in nature (Jennings *et al.*, 2008 ▸). Indeed, the pore system of cement-based materials results from the contribution of several factors, including the interlayer spaces between the layers of C-S-H, for which the width available to water is ≤2 nm, gel pores, with widths of between 2 and 8 nm, capillary pores, with a typical size of 8–10 µm, and macropores, due to deliberately entrained air or inadequate compaction (Kumar & Bhattacharjee, 2003 ▸; Jennings *et al.*, 2015 ▸). It was recently proposed that gel and capillary porosities are the biggest contribution to the total volume, with their proportion varying with ageing and relative humidity (Königsberger *et al.*, 2016 ▸). The use of a 4 µm voxel size instead of 10 µm could help to investigate the capillary pores. Mercury intrusion porosimetry, which is one of the most commonly used techniques to characterize pore structure, has been used on our sample (see supplementary Fig. S1). Although this technique is controversial for cementitious material (Berodier & Scrivener, 2015 ▸; Diamond, 2000 ▸; Stroeven *et al.*, 2010 ▸; Muller & Scrivener, 2017 ▸), the results obtained clearly indicate that using a 4 µm resolution would not, in our case, have improved our understanding of the cement porosity. In addition, the particle sizes before and after hydration are highly heterogeneous. Before hydration, the fly ash, slag and cement have a mean spherical diameter in the range 0.1–100 µm. Of course, their sizes will change during hydration. While the C-S-H will be in the nanometre range (Jennings *et al.*, 2008 ▸), the portlandite will be in the micrometre range (Deschner *et al.*, 2013 ▸). Finally, yet most importantly, the present study focused on the spatial distribution of the carbonation mechanism that occurs in the macropores, for which our resolution was sufficiently high.

### 2.4. XRD-CT data treatment and processing   

The main problem that we encountered during the XRD-CT analysis of the cement paste samples was the huge difference in the size of the crystals in the different phases. In particular, in one of the slices taken from the cured sample, the presence of a single grain of forsterite (a clinker component; Taylor, 1997 ▸) larger than the beam led to spots due to single-crystal artefacts that in turn led to line/streak artefacts in the sinograms after reconstruction (Bleuet *et al.*, 2008 ▸; Vamvakeros *et al.*, 2015 ▸). This problem also occurred for some other voxels, although it was of less significance. To circumvent this issue, we implemented a mathematical treatment (see supporting information) different from those already in the literature (Artioli *et al.*, 2010 ▸; Voltolini *et al.*, 2013 ▸).

### 2.5. Mineralogical identification   

Phase identification was performed using the data obtained for the cement paste that had been cured for six months, in several steps. First, a principal component analysis (PCA) of the volume was made using the *PyMCA* software (Solé *et al.*, 2007 ▸) to determine a series of Bragg peaks that co-varied within the volume, indicating that they were all attributable either to a given phase or to several phases having a similar distribution in the volume. The most intense peaks of each principal component retrieved from the PCA were first qualitatively assigned to those phases that are known to exist in the cement. In the second step, this qualitative assignment was confirmed by calculating the XRD pattern of the phases that were qualitatively identified and checking that all the calculated peaks (and not only the most intense) were present in the experimental XRD pattern. The structure models used for the calculations were those for calcite (Graf, 1961 ▸), ettringite (Moore & Taylor, 1970 ▸), alite (de la Torre *et al.*, 2008 ▸), mullite (Angel *et al.*, 1991 ▸), portlandite (Henderson & Gutowsky, 1962 ▸) and quartz (Levien *et al.*, 1980 ▸). For C-S-H, an experimental pattern was used (taken from a previous study; Grangeon, Claret, Lerouge *et al.*, 2013 ▸). For obvious reasons, we were not able to calculate this for the amorphous component fraction, but it should be noted that its broad diffraction maximum occurs at *d* spacing values similar to those of the *hk* bands at the highest intensity of C-S-H, suggesting that the identified amorphous phase could be ‘proto-C-S-H’ plus unreacted amorphous fractions of FA and BFS. The possibility that this amorphous phase could be amorphous calcium carbonate (ACC) has been ruled out by comparing the experimental XRD pattern with a reference XRD ACC pattern (data not shown).

Once all the phases had been identified, we defined the indicators that could be used to map the spatial distribution of each phase. For the C-S-H and amorphous components, the intensity was summed over a range of *d* spacing values [following Voltolini *et al.* (2013 ▸) for C-S-H, and over the 1.29–1.30 Å range for the amorphous component]. For all the other phases, which were crystalline, the background-subtracted total intensity of a Bragg peak was used. Using the indexing of the structure models given above, this corresponded to the 001, 210, 104, 011 and 100 reflections of, respectively, quartz, mullite, calcite, portlandite and ettringite. In the case of alite, several overlapping peaks were integrated, with the main ones being the 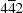, 006 and 440 reflections. A peak at 4.2 Å in the experimental pattern is attributed to the presence of an impurity located outside the polyimide capillary used for the measurement (see Fig. S4).

## 3. Results and discussion   

### 3.1. XRD pattern analysis   

**Figure 2:**
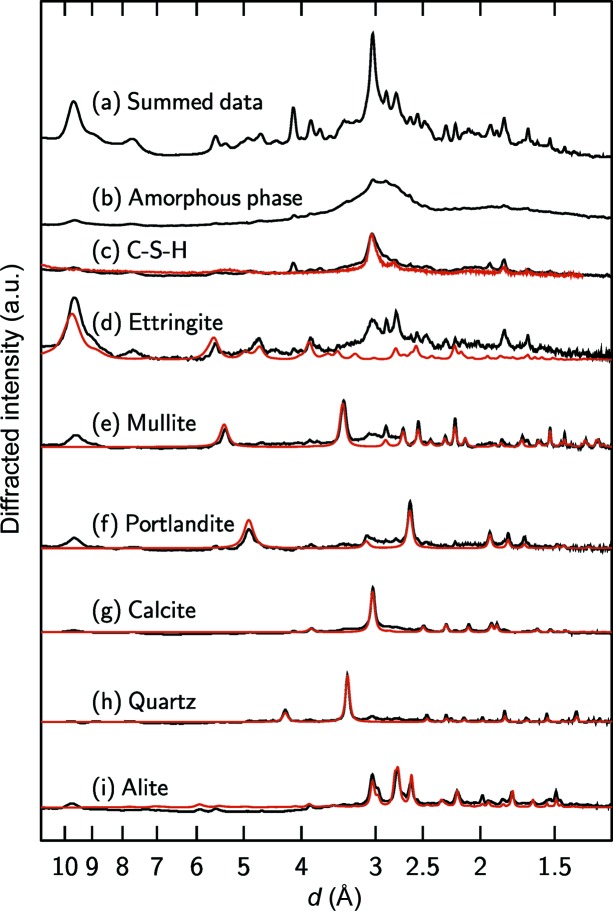
Plots (black lines) representing the experimental XRD patterns (*a*) obtained when summing the whole volume, (*b*) of an amorphous phase (see text for explanation), (*c*) of C-S-H, (*d*) ettringite, (*e*) mullite, (*f*) portlandite, (*g*) calcite and (*h*) quartz. Also included is (*i*) the experimental pattern of alite, which was detected in the *in situ* hydration experiment (see text). The red overlays on the experimental XRD patterns are the corresponding experimental reference pattern or calculated but not refined XRD pattern, and were reproduced according to the published structural models (Angel *et al.*, 1991 ▸; de la Torre *et al.*, 2008 ▸; Graf, 1961 ▸; Henderson & Gutowsky, 1962 ▸; Levien *et al.*, 1980 ▸; Moore & Taylor, 1970 ▸).

**Figure 3:**
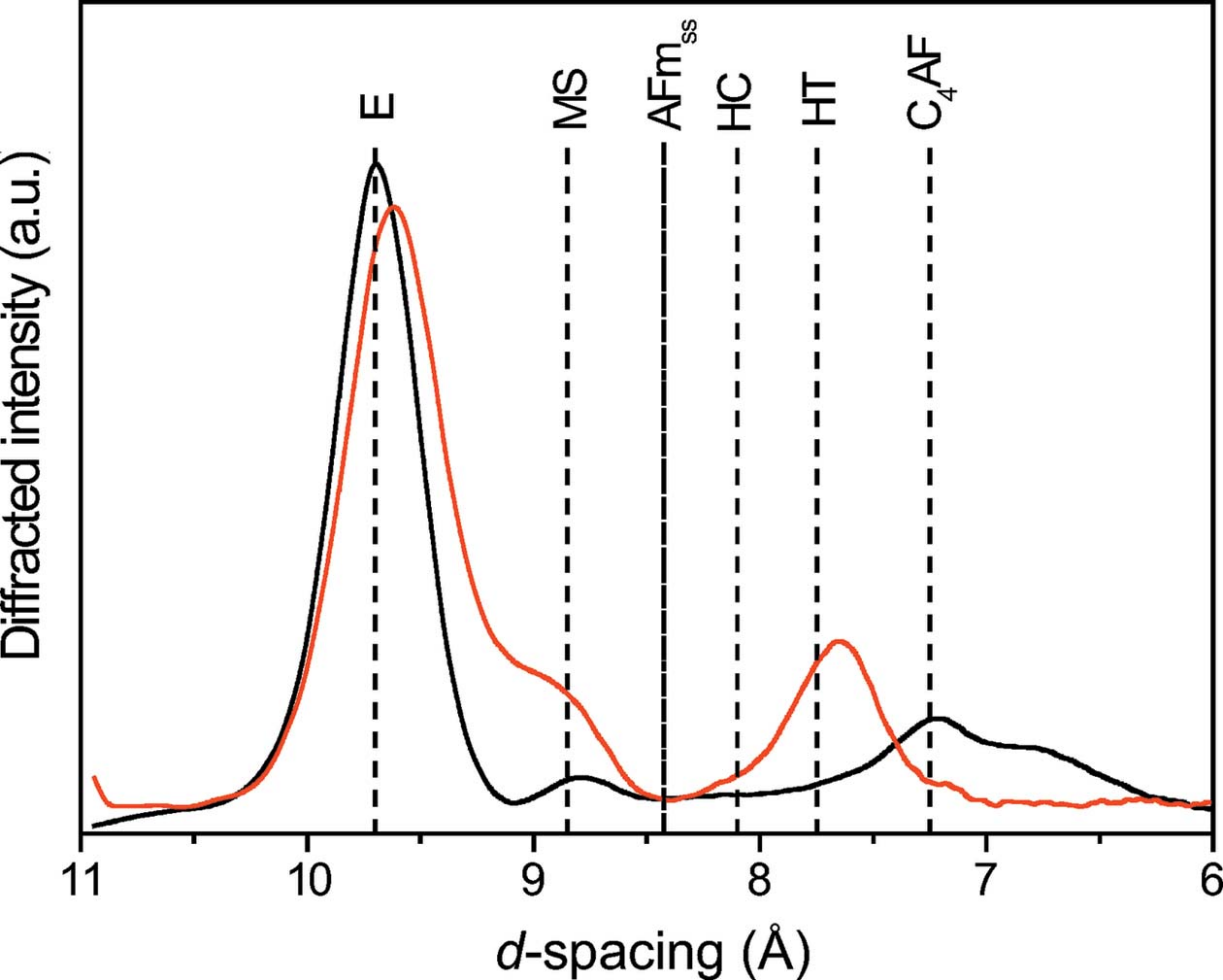
XRD patterns of blast furnace cement after 24 h of hydration (black) and six months of curing (red). E, MS, AFm_ss_, HC, HT and C_4_AF are ettringite, monosulfate, solid solution of hemicarbonate and OH^−^ substituted monosulfate, hemicarbonate, hydrotalcite and ferrite, respectively, as determined by previous studies (Matschei *et al.*, 2007 ▸; Schöler *et al.*, 2015 ▸)

Fig. 2 ▸ shows the experimental and calculated or reference XRD patterns for alite, calcite, C-S-H, ettringite, mullite, portlandite and quartz, which are the major components of the cement paste mineralogy. In addition to these seven phases, we identified an amorphous fraction that also contributed significantly to the overall XRD pattern. For quartz and calcite, we observed very good agreement between the calculated (but not refined) and reference patterns, demonstrating that they were present as pure phases in, at least, the voxels in which their diffracted intensity was the highest. It is worth noting that quartz was observed in a single voxel – in itself, this is a very good demonstration of the technique’s sensitivity. Although a generally very good agreement can be observed between the calculated and reference patterns for the other phases, the 001 maximum of ettringite is present in the experimental patterns of portlandite, mullite, C-S-H and the amorphous phase. This would indicate that ettringite tends to be intimately mixed with all phases. This is further confirmed by the fact that those voxels in which the ettringite diffracted intensity was the highest contained both C-S-H and amorphous phases. Finally, the presence of monosulfoaluminate and hydrotalcite (AFm phases, using the nomenclature of cement chemistry) was determined from a voxel-per-voxel analysis of the high *d* spacing region of the diffraction patterns (Fig. 3 ▸).

### 3.2. Cured ternary blended cement paste mineralogy   

The observed mineralogy, consisting of C-S-H, ettringite, mullite, portlandite, quartz, calcite, an amorphous phase, monosulfoaluminate and hydrotalcite (Figs. 2 ▸ and 3 ▸), is typical of a blended cement (Escalante-García & Sharp, 1998 ▸; Hill & Sharp, 2002 ▸). Portlandite, C-S-H and ettringite are produced by the hydration of clinker (Lothenbach, 2010 ▸; Scrivener *et al.*, 2004 ▸), while mullite is present in the reactive blender, being a component of silicoaluminous fly ash (Alahrache *et al.*, 2016 ▸; Gomes & François, 2000 ▸) or pulverized fuel ash (Escalante-García & Sharp, 1998 ▸; Hill & Sharp, 2002 ▸). Typically, studies that focused on the hydration of binary Portland cement blends used either fly ash (FA) or silica fume (SF) (Vollpracht *et al.*, 2016 ▸). In these systems, the presence of portlandite, C-S-H, ettringite, hydrotalcite, ferrite, monocarbonate/stratlingite and unreacted FA or SF is expected according to the thermo­dynamic calculations (Vollpracht *et al.*, 2016 ▸). In addition, both thermodynamic modelling and XRD patterns obtained after hydration of a ternary Portland cement blend [blast furnace slag (BFS) 20% and FA 30% composition] for six months, which was very similar to the cement blend used in the present study, evinced the presence of monocarbonate, ettringite, C-S-H, portlandite, hydrotalcite, and amorphous BFS and FA. Regarding the AFm and AFt phases, ettringite was the dominant phase, consistent with our results. In addition to ettringite, we detected monosulfoaluminate and hydrotalcite (Fig. 3 ▸). The presence of hemicarbonate cannot be ruled out but the signal was weak. The solid solutions of hemicarbonate and OH-substituted monosulfate reported in some studies (Matschei *et al.*, 2007 ▸; Schöler *et al.*, 2015 ▸) were not detected here. The presence of calcite is not expected in a preserved cement paste, so its presence in the sample certainly resulted from the carbonation of the portlandite or C-S-H (Ruiz-Agudo *et al.*, 2013 ▸), caused by the sample coming into contact with the atmosphere during preparation and storage. Finally, the amorphous phase detected here was not C-S-H, which produces well defined though broad diffraction maxima (Grangeon, Claret, Lerouge *et al.*, 2013 ▸; Grangeon, Claret, Linard & Chiaberge, 2013 ▸; Grangeon *et al.*, 2016 ▸, 2017 ▸), but may be a ‘proto-C-S-H’ according to the discussion above, or remnants of FA or BFS (Schöler *et al.*, 2015 ▸). The fact that the XRD patterns (Fig. S5) obtained from the inside to the outside of the amorphous phase grain look very similar to those of C-S-H suggests that the ‘proto-C-S-H’ hypothesis may be relevant. The distinction we make between C-S-H and the rest of the amorphous matrix is seldom made in XRD studies, due to the poor crystalline character of this phase (Scrivener *et al.*, 2004 ▸; Voltolini *et al.*, 2013 ▸), but was possible in this case due to the high data signal-to-noise ratio. The presence of an amorphous phase at a later stage is kinetically driven and may have been linked here to the lack of water-filled capillary pores, resulting in a lower degree of slag and fly ash reactions (Berodier & Scrivener, 2015 ▸).

### 3.3. Spatial distribution of different phases   

**Figure 4:**
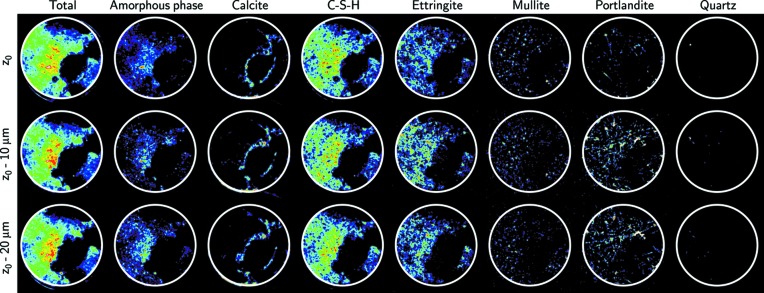
Distribution of the phases in the sample cured for six months. From left to right, the distribution maps integrate the contribution of all the identified phases, the amorphous phase, calcite, the C-S-H plus the calcite and the amorphous phase, and the ettringite, mullite, portlandite and quartz phases. Red values indicate a higher content of the phase in the distribution map. Black values indicate voids (Total map) and voids or absence of phase (all other maps). The typical ratio of counts for black values to red values is 0.2. Because all the maps were constructed from the number of counts integrated according to the procedure described in the *Materials and methods* section[Sec sec2], they were scaled individually, and the sum of the colours for each map for the individual phases does not equal the colour of the total intensity map. The three slices were collected, from top to bottom, at vertical positions *z*_0_ (arbitrarily chosen in the sample), 10 µm below *z*_0_ and 20 µm below *z*_0_. As the beam size was 10 µm, the slices collected at *z*_0_ and *z*_0_ − 10 µm are thus adjacent in the sample, as are those collected at *z*_0_ − 10 µm and *z*_0_ − 20 µm. The white circle of 1.6 mm diameter corresponds to the limit of the sample.

As illustrated by the two-dimensional distribution maps (Fig. 4 ▸), the total diffracted intensity varies within the volume, indicating heterogeneous mineralogy and/or density. Those voxels with a diffracted intensity close to zero were assumed to be voids. A previous report (Collins & Sanjayan, 2000 ▸) stated that voids and microcracks (due to both entrapped air and cracks) account for about 6% of the volume in alkali-activated slag and ordinary Portland cements. Here, potential microcracking arising from the drilling of the sample as well as the microporosity (Jennings *et al.*, 2015 ▸) could not be identified because of our voxel size resolution. In contrast, the observation of the presence of macroporosity was straightforward.

### 3.4. Carbonation development and localization   

**Figure 5:**
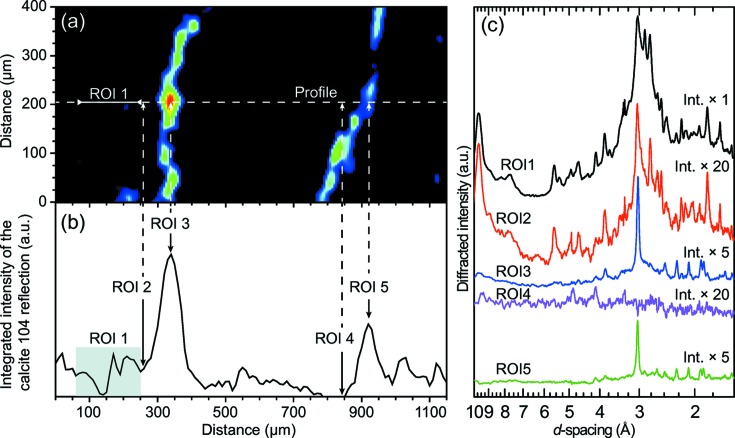
(*a*) Mapping of the integrated intensity of the calcite 104 reflection. The area is the same as the central part of the porosity shown in Fig. 4 ▸, slice *z*_0_. The white dashed line represents the profile along which the calcite 104 reflection has been integrated [see panel (*b*)]. (*c*) The XRD patterns reconstructed for the five regions of interest (ROI) identified in panel (*b*) are plotted. Note that the XRD patterns collected in ROIs 3 and 5 are pure calcite (see Fig. 3 ▸). The width of the ROI is 1 pixel (10 µm), with the exception of ROI 1 which is larger. The number after Int. × indicates the multiplication factor used to scale the intensity in the plot.

Calcite was found to be segregated at the interface between the cement and macropores (Fig. 5 ▸). To quantify the depth of this carbonation phenomenon, the intensity of the calcite 104 reflection was integrated along a profile (Figs. 5 ▸ *a* and 5 ▸ *b*) and it was found that the carbonation was limited to the first 100 µm at the interface, thus providing a robust estimate for the carbonation depth around the macropores that are more likely air-entrapped macropores. To confirm that those macropores are not capillary gel–water pores, ptychographic X-ray CT should be used (Cuesta *et al.*, 2017 ▸). An examination of the XRD patterns selected in different regions of interest (ROIs) showed that the ROIs located next to the porosity had an XRD pattern that matched that of the calcite (Fig. 5 ▸ *c*), whereas in the inner part of the sample calcite could not be detected. This shows that the area subjected to carbonation consists only of calcite, and that other calcium carbonate polymorphs like vaterite or aragonite are not present. These observations are compatible with previous findings (Morandeau & White, 2015*a* ▸,*b* ▸) concerning carbonation processes, especially given that the CEM V blended cement used in this study is known to be sensitive to the carbonation process (Lagosz & Deja, 2012 ▸), certainly because the SF and FA increase the carbonation depth (Singh & Singh, 2016 ▸). Finally, the carbonation had a greater effect on the larger pores than on the smaller ones (Pihlajavaara, 1968 ▸). In contrast with calcite, other phases were found to be reasonably evenly distributed throughout the entire volume, with the exception of quartz for which only a single grain could be identified (one voxel on the upper right-hand side of the sample, Fig. 4 ▸).

### 3.5. *In situ* XRD-CT time-resolved hydration   

In addition to examining the blended cement that had been cured for six months, XRD-CT was also carried out on the binder after 24 and 30 h of hydration. Measuring the earlier stages of hydration proved practically impossible with the current experimental setup, as the mineralogy changes occurring during the first hours of cement hydration were faster than the measurement time required for the acquisition of a single slice.

**Figure 6:**
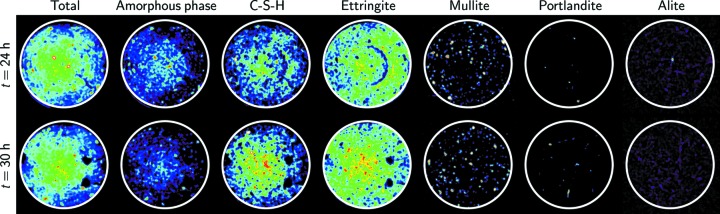
Distribution of the phases after (top row) 24 h and (bottom row) 30 h of cement hydration. From left to right, the distribution maps integrate the contribution of all the identified phases and the amorphous, C-S-H, ettringite, mullite, portlandite and alite phases. Red values indicate a higher content of the phase in the distribution map. Black values indicate voids (Total map) and voids or absence of phase (all other maps). Because all the maps were constructed from the number of counts integrated according to the procedure described in the *Materials and methods* section[Sec sec2], they were scaled individually, and the sum of the colours of each map for the individual phases does not equal the colour of the total intensity map. The white circle of 1.3 mm diameter corresponds to the limit of the sample.

Most of the phases identified in the cement that had been cured for six months were already present after 24 h of hydration (see Fig. S3). In addition to the previously reported phases, Fig. 6 ▸ also shows those phases that were not detected in the former and those undergoing significant changes with time, from 24 to 30 h of hydration time. Note that, because of their scarcity, these phases could not be detected in the averaged pattern, and were thus identified based on a voxel-to-voxel analysis. Within these two measurement steps, the main changes were the development of ettringite and C-S-H, the decrease in the amount of amorphous matrix and alite, and the development of macroporosity in response to the intrinsic nature of the cement hydration process. Indeed, with an increase in the hydration time, the porosity related to autogenous shrinkage, due to the surface tension within the capillaries created by water demand during the hydration process, also increases (Königsberger *et al.*, 2016 ▸). Again, concomitant with ettringite formation, monosulfoaluminate and hydrotalcite were formed (Fig. 3 ▸).

Finally, a detailed comparison between the high *d* spacing side of the XRD patterns of those samples aged for 24 h and six months allowed subtle changes to be detected. In particular, a diffraction maximum attributable to ferrite was observed in the sample aged for 24 h, but this could not be detected in the sample aged for six months (Fig. 3 ▸). This observation, associated with the observed almost-complete hydration of alite in less than 30 h, implied a rather fast hydration. This may seem to be inconsistent with a previous study of Portland cement hydration using Rietveld analysis, which showed a fast alite hydration within the first few days but its persistence over several months (Scrivener *et al.*, 2004 ▸). However, we should remember that only three slices were examined by XRD-CT and that some alite may remain at the sample scale. In addition, the hydration of alite is considerably accelerated due to the admixture of nanosilica (Land & Stephan, 2012 ▸) that can be provided by the FA in our case.

## 4. Conclusions   

Using XRD-CT measurements, we have resolved the cement mineralogy both non-invasively and *in situ* as a function of hydration time. The observed mineralogy is consistent with that described in the literature for a dilute system. In comparison with those studies that used this technique on well crystallized systems (Vamvakeros *et al.*, 2015 ▸), it is not easy to analyse the collected signal but relevant information can still be gathered. For example, thanks to a very high signal-to-noise ratio within the probed voxel size, we could locate the carbonation with high accuracy. This is one of the major factors causing structural deterioration. The results described in this article have broader implications for understanding in a non-invasive manner the complex mineralogical paragenesis of concrete structures exposed to aggressive environments. Furthermore, recent improvements in beam focusing at the sub-micrometre level will allow the spatial location of these heterogeneous reaction products.

## Supplementary Material

Additional information and figures. DOI: 10.1107/S205225251701836X/ro5009sup1.pdf
