# Supplementary material for: Deciphering mineralogical changes and carbonation development during hydration and ageing of a consolidated ternary blended cement paste
Source: IUCrJ. 2018 Jan 19;5(Pt 2):150–7. doi: 10.1107/S205225251701836X (PMC5947719; doi:10.1107/S205225251701836X)
Supplement: Supplementary file 1 [file m-05-00150-sup1.pdf]

IUCrJ

Volume 5 (2018)

Supporting information for article:

Deciphering mineralogical changes and carbonation development during hydration and ageing of a consolidated ternary blended cement paste

Francis Claret, Sylvain Grangeon, Annick Loschetter, Christophe Tournassat, Wout De Nolf, Nicholas Harker, Faiza Boulahya, Stéphane Gaboreau, Yannick Linard, Xavier Bourbon, Alejandro Fernandez-Martinez and Jonathan Wright

S1. Composition of ternary blend cement

The composition of the ternary blend cement is given in Table S1. In addition to the main binder constituents indicated in the Table, calcium sulfate, organic additives and a reducing agent are introduced in small quantities.

Table S1 Composition of CEM V/A (S-V) 42.5 N CE PM-ES-CP1 NF "PMF3"

Oxide composition (wt%)		Main binder constituents (wt%)		Constituent characteristics	
SiO_2	29.4	Couvrot's clinker	54	CaO/SiO_2	3.1
Al_2O_3	10.2			C3S (%)	67
Fe_2O_3	3.3			C2S (%)	12
TiO_2	0.5			C3A (%)	11
MnO	0.2			C4AF (%)	7
CaO	47.3			MgO (%)	1
MgO	2.7				
SO_3	2.9	BFS**	23	$(CaO+ MgO)/SiO_2$	1.4
K_2O	1.42			$CaO+ MgO) + SiO_2$	83.2
Na_2O	0.23				
P_2O_5	0.2	FA***	23	Reactive CaO (%)	0.9
Cl^-	0.21			Reactive SiO_2 (%)	44.2
S^{2-}	0.02			LOI (%)	4.5
LOI*	2				

* LOI: Loss on ignition (the weight lost when the cement was heated to 1000˚C)

**BFS: Blast furnace slag

***FA: Fly ash

S2. Mercury intrusion porosimetry measurement

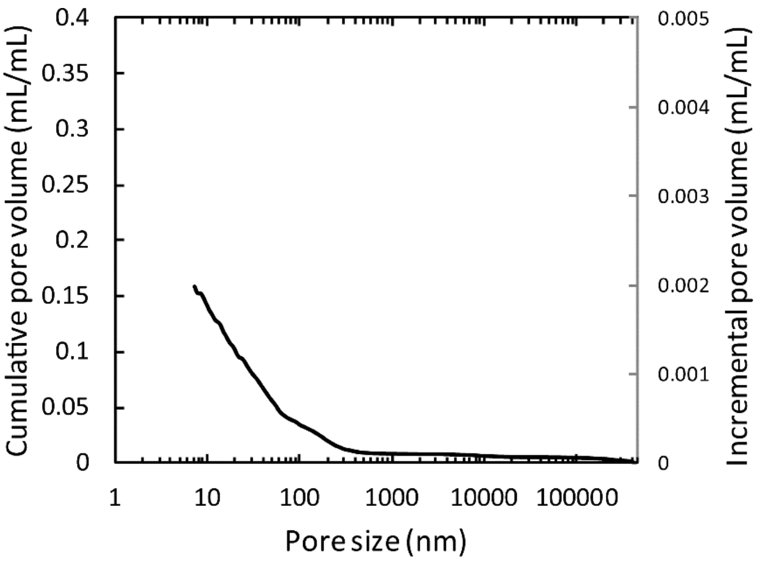

Figure S1 Cumulative intrusion curve and incremental throat size distribution for CEM V as measured by MIP.

S1. XRD-CT data treatment and processing

S1.1. *Spot removal*

Large crystals lead to 'spotty' diffraction images (Figure S2.A). Filtering approaches to deal with single-crystal artefacts in XRD-CT already exist (Vamvakeros et al., 2015). In the present study, we used a routine based on the following two concepts to process the raw data and provide smooth rings which have a non-uniform intensity distribution. The stepwise approach was as follows:

i. First, identify pixels affected by spots due to preferential orientation. We used a relative intensity criterion based on a comparison with the intensity of the neighbouring pixels.

ii. Second, correct the intensity of the pixels identified as being affected by the presence of large grains.

It is therefore necessary to work ring by ring, in a stepwise approach. The raw image and the mask were first corrected from distortions arising from the instrumental set up using the "pyFAI.distortion.correct" function (Ashiotis et al., 2015). A distance matrix was then created (one per image stack), that attributes, to each pixel, its distance to the circles' centre. A loop then analyses the pixels ring by ring, considering those pixels between a distance r and a distance r + p1, where p1 is a setup parameter. Here, the best compromise between the computation time and treatment

accuracy was reached with a value of p1 = 3 pixels. In a second step and for each ring, degraded pixels are identified by comparing their intensity with that of the median ring, multiplied by a factor of p2. A value of 1.04 was determined to be the most appropriate. All the pixels that exceed this value were deemed to be degraded, and were replaced by the median value. An example processed image is shown in Figure S2.B. This mathematical treatment costs less than half a day of computing for a full stack of images when using 60 nodes of a high-performance supercomputer. Obviously, it was not possible to visually inspect all the images (30,000 images per sample); however, control was applied both to the number of random images in the sample, and to the images with extreme characteristics in terms of standard deviation and average intensity. After applying the “pyFAI.distortion.uncorrect” function, the stack of processed images was further treated with common pyFAI functions and a sinogram was produced (Figure S2.C). The following defect was visible in the resulting sinogram: a vertical average intensity discrepancy, characterised by horizontal lines of useless pixels, and by an abrupt change in the average intensity on the horizontal lines above and below the faulty lines. These are due to beam shutdown and refill during the measurement.

We analysed the discrepancy in the vertical average intensity and corrected it by applying an appropriate linear function to one of the horizontal compartments. The faulty lines were replaced by a weighted average of valuable lines above and below them, with weights depending on the distance between lines (Figure S2. 2D). A single grain of forstierite, a clinker component (Taylor, 1997) that gave a high intensity curve, (Figure S2. 2C) was eliminated because it oversaturated the signal.

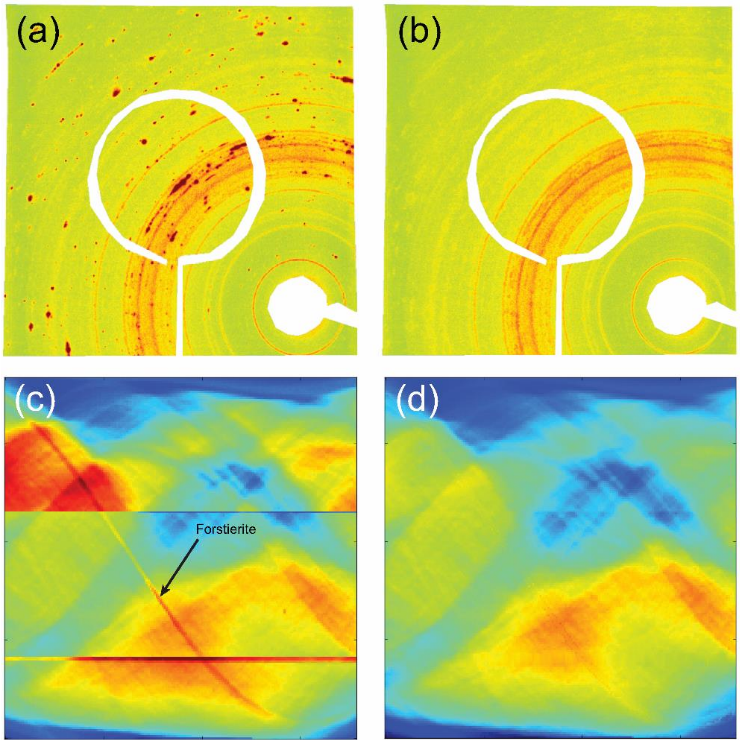

Figure S2 (a): Raw image acquired for a given voxel, before any processing or interpretation. (b): Same image after processing to remove the saturated pixels. The white areas correspond to the useless image pixels that are ignored (through the application of a mask) in the subsequent steps. (c): Raw sinogram. (d): Same sinogram after processing to remove the vertical intensity changes.

S1.2. Average XRD patterns

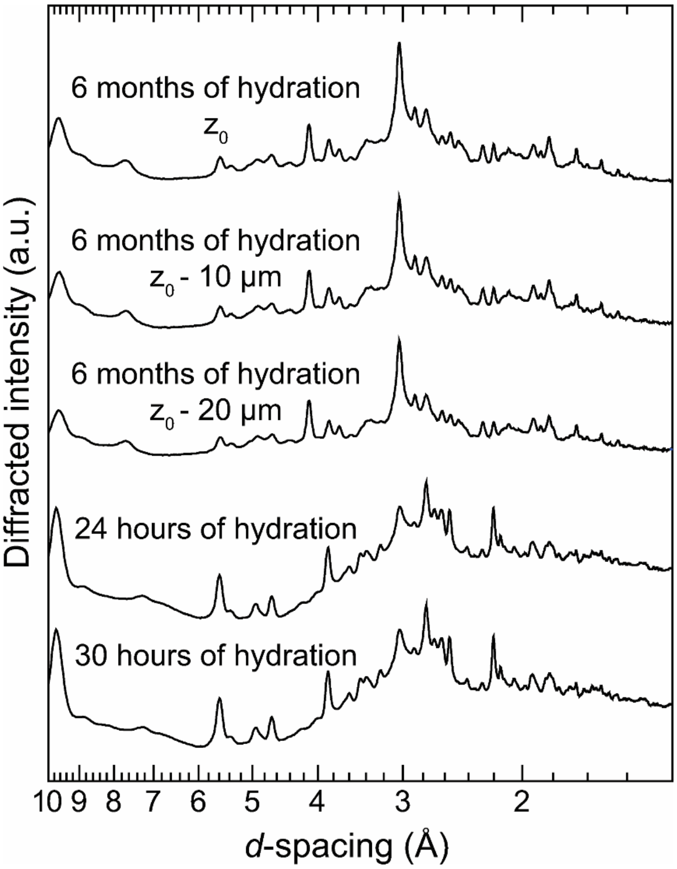

Figure S3 XRD experimental diffraction patterns obtained when summing the whole volume both for the cured cement and after 24 and 30 h of hydration. The data for the sample cured for six months were recorded for three slices. They were collected, from top to bottom, at vertical position z_0 (arbitrarily chosen in the sample), 10 µm below z_0, and 20 µm below z_0. As the beam size was 10 µm, the slices collected at z0 and z0 - 10 µm were adjacent in the sample, as were those collected at z_0 - 10 µm and z_0 - 20 µm.

S2. Impurity outside polyimide capillary used for the measurement

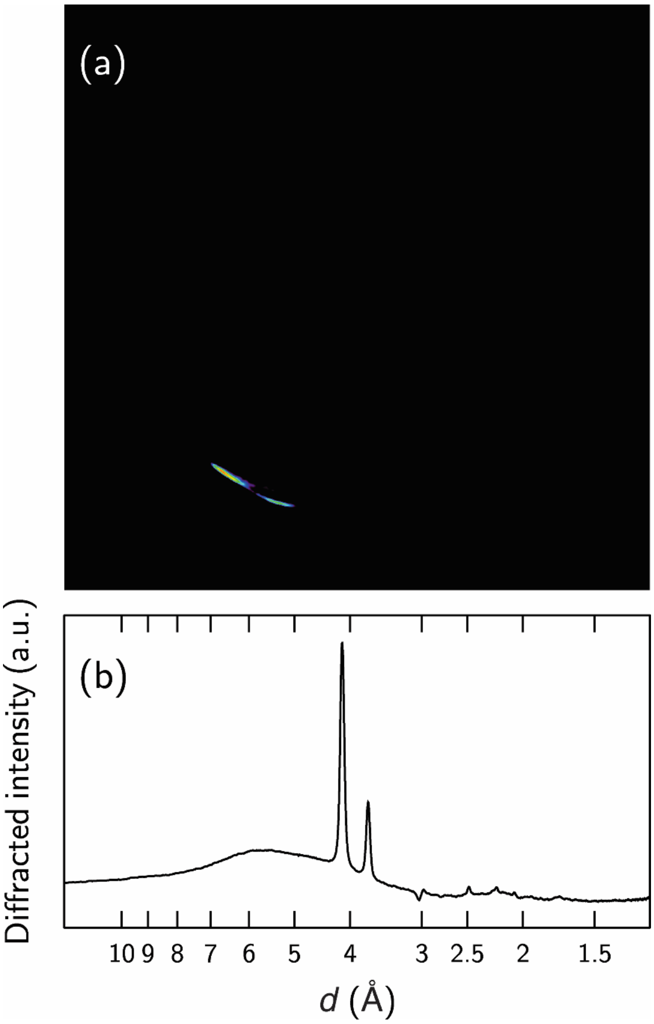

Figure S4 Shows an impurity localised outside the polyimide capillary (a) and the corresponding XRD experimental diffraction pattern (b).

S3. Proto C-S-H

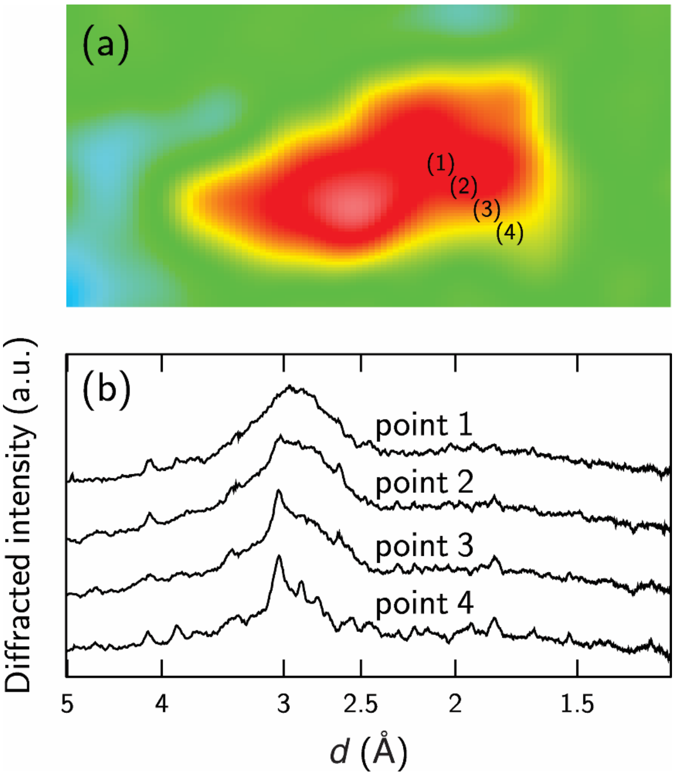

Figure S5 Top: Enlarged view of area in which amorphous rich phase (approximately 80 x 150 μm) is present. Bottom: XRD experimental diffraction patterns obtained from inner part of the phase to the outside (1 to 4, respectively).
